# Profiling of human burned bones: oxidising *versus* reducing conditions

**DOI:** 10.1038/s41598-020-80462-3

**Published:** 2021-01-14

**Authors:** M. P. M. Marques, D. Gonçalves, A. P. Mamede, T. Coutinho, E. Cunha, W. Kockelmann, S. F. Parker, L. A. E. Batista de Carvalho

**Affiliations:** 1 Molecular Physical-Chemistry R&D Unit, Department of Chemistry, University of Coimbra, 3004-535 Coimbra, Portugal; 2 Department of Life Sciences, University of Coimbra, 3000-456 Coimbra, Portugal; 3 Laboratory of Forensic Anthropology, Centre for Functional Ecology, University of Coimbra, 3000-456 Coimbra, Portugal; 4 Research Centre for Anthropology and Health (CIAS), University of Coimbra, 3000-456 Coimbra, Portugal; 5 Archaeosciences Laboratory, Directorate General Cultural Heritage (LARC/CIBIO/InBIO), 1349-021 Lisbon, Portugal; 6 ISIS Facility, STFC Rutherford Appleton Laboratory, Chilton, Didcot, OX11 0QX UK

**Keywords:** Infrared spectroscopy, Raman spectroscopy, Archaeology, Biological anthropology

## Abstract

Complementary optical and neutron-based vibrational spectroscopy techniques (Infrared, Raman and inelastic neutron scattering) were applied to the study of human bones (femur and humerus) burned simultaneously under either aerobic or anaerobic conditions, in a wide range of temperatures (400 to 1000 °C). This is the first INS study of human skeletal remains heated in an oxygen-deprived atmosphere. Clear differences were observed between both types of samples, namely the absence of hydroxyapatite’s OH vibrational bands in bone burned anaerobically (in unsealed containers), coupled to the presence of cyanamide (NCNH_2_) and portlandite (Ca(OH)_2_) in these reductive conditions. These results are expected to allow a better understanding of the heat effect on bone´s constituents in distinct environmental settings, thus contributing for an accurate characterisation of both forensic and archaeological human skeletal remains found in distinct scenarios regarding oxygen availability.

## Introduction

Human burned bones are often the only remains found in forensic scenarios (*e.g.* from terrorist attacks, explosions or fires) and archaeological settings, from which the bioanthropologists aim to identify victims or obtain information on past populations (*e.g.* age at death or details of funerary practices), particularly regarding the use of pyrotechnology. In order to achieve this goal, it is essential to understand the effect of the heating conditions on the skeletal remains, with a view to accurately characterise the resulting structural and chemical changes and ascertain the parameters that have shaped the bone. These heat-elicited variations are dependent on the temperature and duration of heat exposure, on the burning environment (namely oxygen availability and presence of metals or organic materials in the surroundings) and also on the morphology of the bone (*e.g.* ratio of compacta to spongiosa structures)^1–4^. Two main types of burning can be distinguished: in the presence of oxygen (combustion, oxidising conditions) or in the absence of oxygen (reducing conditions).

The analysis of burned bones is not straightforward due to the significant heat-prompted changes experienced by the samples, at both the macroscopic (chemical composition, colour and dimensions) and microscopic (crystallinity variations) levels^1,2,5–14^. These interfere with the reliability of most anthropological analytical methods, which are based on references from unburned bones. Also, the bone retains its original biological features only up to a certain degree of burning. DNA analyses, for instance, are dependable solely at low to medium temperatures (250–400 °C)^15–17^, although DNA has been retrieved from bones subject to heat exposures up to *ca.* 600 °C but with a high risk of contamination and degradation^18,19^. Additionally, the temperature to which a bone has been subject cannot be determined exclusively according to macroscopic features (such as colour), since these can vary even for the same heating temperature (according to the surrounding conditions).

In the last two decades vibrational spectroscopy techniques—Fourier transform infrared (FTIR), Raman and more recently inelastic neutron scattering (INS)—have been shown to be useful tools for the identification and characterisation of burned skeletal remains (faunal and human) from both forensic and archaeological scenarios^6,7,20–24^. These techniques allow the assessment, with high accuracy and sensitivity, of the diagenesis-induced chemical alterations in bone, particularly those prompted by burning events (essentially under oxidising conditions): loss of organic components (collagen and lipids), carbonate depletion, and phosphate and hydroxyl rearrangements within the bone´s inorganic framework. Raman spectroscopy, in particular, is a method of choice for the detection of organic constituents (*e.g.* collagen)^25^, despite the drawback of bone´s strong fluorescence for samples subject to temperatures below 600–700 °C. FTIR, often using the attenuated total reflectance (ATR) mode (which avoids any type of sample preparation), has become the most commonly applied non-invasive vibrational spectroscopy tool for analysing skeletal remains, both in forensic^5,22,26^ and archaeological^23,24,27–30^ sciences. INS, in turn, is an extremely useful technique for probing a hydrogenous material such as bone, the intensity of each vibrational transition being expressed, for a given atom, by the dynamic structure factor

1 $${\mathbf{S}}_{\mathbf{i}}^{*}\left(\mathbf{Q},{\nu}_{\mathbf{k}}\right)= \frac{\left({\mathbf{Q}}^{2}{\mathbf{u}}_{\mathbf{i}}^{2}\right){\sigma}}{3}\mathbf{e}\mathbf{x}\mathbf{p}\left(-\frac{{\mathbf{Q}}^{2}{{\varvec{\upalpha}}}_{\mathbf{i}}^{2}}{3}\right)$$

where $\mathrm{Q }(\mathrm{\AA }$^−1^) is the momentum transferred to the sample, ${\nu}_{\mathbf{k}}$ is the energy of a vibrational mode, ${\mathrm{u}}_{\mathrm{i}}(\mathrm{\AA }$) is the displacement vector of atom *i* in mode *k*, σ is the neutron scattering cross section of the atom, and ${\mathrm{\alpha }}_{\mathrm{i}}(\mathrm{\AA })$ is related to a mass-weighted sum of the displacements of the atom in all vibrational modes. In contrast to FTIR and Raman there are no selection rules for INS, which allows observation of all the fundamental vibrational modes, overtones and combination bands for the samples under study. A particular advantage is that the very low energy range (0–400 cm^−1^) is readily accessible by this technique. It therefore enables the detection of transformations within bone´s inorganic matrix associated with alterations in microcrystallinity, revealing any changes in the H-bond pattern that are prone to occur upon heating events^31^.

The development of a method able to detect and quantify diagenetic changes in human burned skeletal remains, beyond the macroscopically visible manifestations, has been pursued by the authors through the use of vibrational spectroscopy (both optical and neutron-based) coupled with diffraction methods (X-ray and neutron diffraction). This included a pioneering approach focusing on the intrinsic properties of bone (microcrystalline structure), based on the analysis of human bones burned under controlled conditions by inelastic neutron scattering spectroscopy^13,30,32–34^. Up to 2016 only a limited number of INS studies of bone had been reported^35–37^ and none of samples subject to heating, despite the usefulness of INS for the characterisation of this type of tissue particularly regarding its inorganic network (hydroxyapatite, HAp, Ca_10_(PO_4_)_6_OH_x_). The studies performed by the authors, using a combined spectroscopic (FTIR, Raman, INS)^11,13,30,32–34,38^ and neutron diffraction approach^31^, have allowed the identification of distinctive features such as changes in organic components´ and carbonate content, chemical substitutions at the hydroxyl sites and heat-elicited structural variations, in both forensic and archaeological skeletal remains. These revealed even minor differences in bone composition and have allowed us to develop a *chemosteometric* regression model that quantitatively relates specific infrared spectral biomarkers to heat-induced metric changes, for each burning temperature^39^. Therefore, this approach may currently be applied to estimate the pre-burning metric dimensions of bones which, in turn, can for example be used to make inferences about sex estimation.

Following these successful studies, the present work aims to complement the results obtained so far with vibrational spectroscopic data measured on bones burned under different conditions—regarding temperature and oxygen availability—with a view to attain a detailed interpretation of the chemical and crystallinity variations undergone by the samples as a function of the surrounding conditions. Human bone samples (from femur and humerus) were probed after aerobic burning (combustion) or anaerobic burning (either in a sealed chamber or in an unsealed container allowing venting of volatiles), under experimentally controlled conditions, at different maximum temperatures (from 400 to 1000 °C). To the best of the authors´ knowledge, this is the first study of human burned bone in an oxygen-deprived setting (within a wide temperature interval) by complementary optical and neutron vibrational spectroscopy techniques. We note that a FTIR and Raman study of burned bovine bone has been reported by Reidsma and coworkers^21^, that evidences distinct heat-induced alterations in bone under oxidising or reducing atmospheres.

The results obtained are expected to contribute to a more accurate identification of skeletal remains subject to unknown heating events, by providing information on the environmental parameters at the time of burning, namely the type of atmosphere and the temperature and duration of the burning. Indeed, there is a high variability in the conditions around the body during the heating process—temperature, indoor or outdoor burning, oxygen availability, use and type of fuels, soil composition or body wrapping. This is particularly important for the characterisation of skeletal remains found in forensic and archaeological settings (that may comprise specimens burned both with and without oxygen), through reconstruction of the heating conditions (*e.g.* funerary practices such as cremation, pyrotechnological manufacturing of bone artefacts, accidents involving explosions and fire, or criminal burning of victim´s corpses).

## Materials and methods

### Materials

The bones currently analysed were obtained from a human skeleton (skeleton 42 from the cemetery of Capuchos, Santarém, Portugal (CC_NI_42)) belonging to a collection of unidentified human skeletons donated for research purposes, which is housed at the Laboratory of Forensic Anthropology of the University of Coimbra^40^. The individuals were buried for at least three years, which is the minimum required period of inhumation time prior to exhumation according to Portuguese legislation (Decreto-Lei 411/98). The age at death and sex of individual CC_NI_42 are unknown. Samples were collected from a femur and a humerus. No replicates were used due to limited sample resources—a human skeletal collection as rare as this must be preserved (without compromising scientific advances). The use of two different bones from the same skeleton was chosen to detect potential replicability problems. Authorization for research on this collection was granted by the Ethics Committee of the Faculty of Medicine of the University of Coimbra (reference number: CE_026.2016).

The highly crystalline SRM 2910b calcium hydroxyapatite (HAp, Ca_10_(PO_4_)_6_(OH)_2_, Ca/P = 1.67) from NIST (Gaithersburg/MA, USA)^41^ was used as a reference material (crystallinity index = 7.91, as compared to 3.79 for commercial HAp), both as received and after heating to 1000 °C (anaerobically, in an unsealed container).

### Sample preparation and controlled bone burning

Thirty-six samples were used in this study—eighteen from the femur and eighteen from the humerus. Bone sections of *ca.* 2 cm in length from the femur and the humerus were cut from skeleton 42 with a DREMEL mini-saw electric tool. These bones were completely devoid of soft tissue and marrow, and were neither dehydrated nor degreased before spectral analysis. Contaminants from the outer layer were removed by gentle sanding.

**Figure 1:**
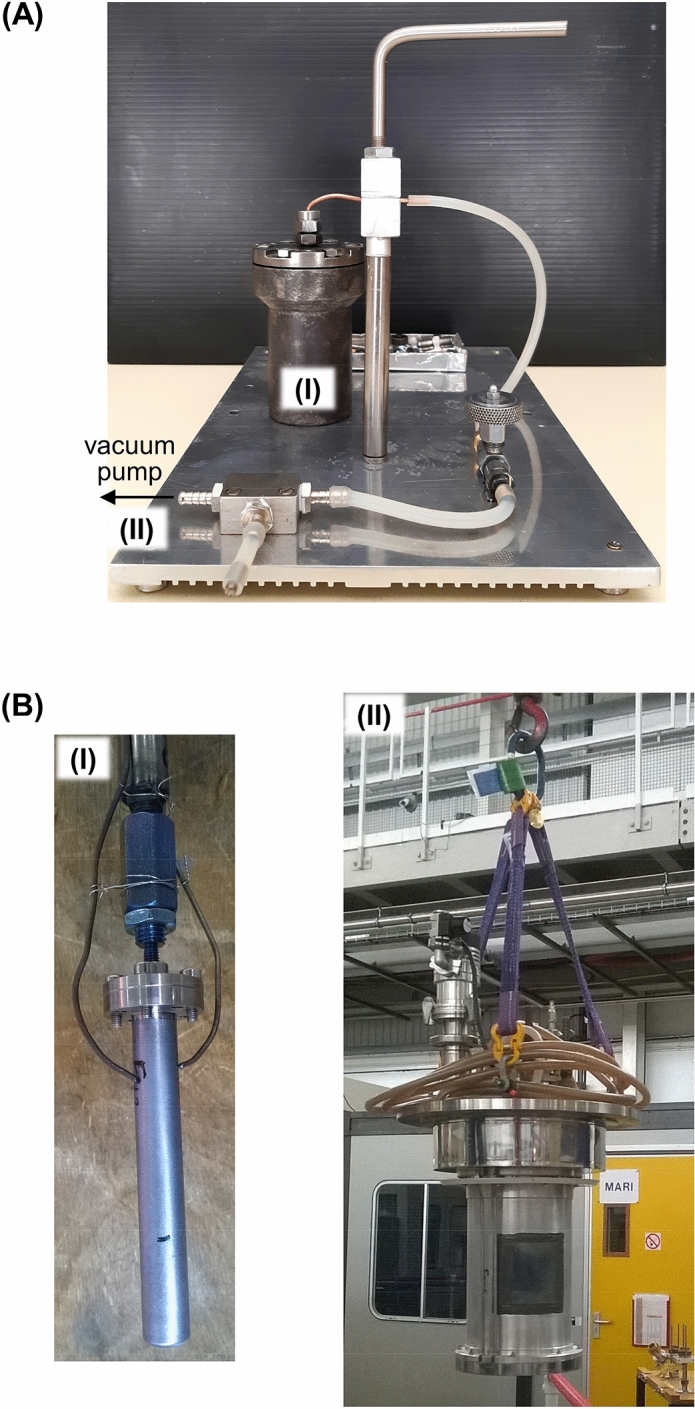
Experimental setups for the burning process of human bone samples under controlled anaerobic conditions: (**A**) An_sealed_—each bone fragment was placed inside a home-made steel airtight chamber (I) which was vacuum-pumped (II) and then inserted into an electric muffle furnace for heating at defined temperatures (at the University of Coimbra, Portugal); (**B**) An_unsealed_—each powdered bone sample was placed into a vanadium container (I) with a perforated lid (allowing volatiles venting), that was inserted into the instrument furnace (II), under vacuum, and heated at defined temperatures (GEM instrument, at the ISIS Facility, United Kingdom).

Adjoining samples of the same bone were simultaneously burned aerobically (n = 18) and anaerobically (n = 18), at the University of Coimbra (Portugal). For the latter, the bone fragments were placed in a home-made steel airtight chamber, which was vacuum-pumped (< 1 mBar). This chamber was then placed inside an electric muffle furnace (BARRACHA model K-3, three-phased, 14 A, 43 × 43 × 56 cm internal dimensions, manufactured by Barracha Lda., Leiria, Portugal, 2012) coupled to an automatic programmer with a digital temperature indicator, allowing programmed start-up and automatic heating speed variation (Fig. 1A). A type K thermocouple (negative/nickel-aluminium, positive/nickel-chrome), following norm IEC 60584-2, was used to measure the temperature inside the muffle furnace. The samples were pooled into groups of four, and the resulting nine groups were burned under controlled conditions—therefore, each experiment included one femur and one humerus fragments aerobically burned, as well as one femur and one humerus samples anaerobically burned. The following maximum temperatures and durations were applied: 400 °C (120 min), 500 °C (120 min), 600 °C (120 min), 700 °C (120 min), 750 °C (120 min), 800 °C (134 min), 850 °C (167 min), 900 °C (181 min) and 1000 $^\circ$ C (221 min). These burning times refer to the period needed to attain each maximum temperature, after which the muffle furnace was immediately switched off. The samples were left to cool to room temperature before removing them from the furnace. Each fragment of burned bone was ground and sieved (to a mesh size of 400 μm), yielding 2 to 5 g.

Anaerobic burning in an unsealed container was performed at the ISIS Facility (Didcot, United Kingdom) (on the General Materials instrument)^31^—powdered samples of each bone fragment were placed in vanadium containers of 11 mm diameter and 0.15 mm wall thickness, with a perforated lid allowing venting of volatiles. Each container was inserted into a furnace, which was evacuated to < 10^–3^ mbar and heated up to 1000 °C in steps of 100 °C, at a heating rate of 100 $^\circ$C/min and with a holding time of 1 h at each step. The temperature of the vanadium container was monitored with two type K thermocouple sensors (Fig. 1B).

Overall, three sets of experiments were carried out yielding three groups of bone samples: (i) aerobically burned (oxidising conditions); (ii) anaerobically burned (reducing conditions) in an unsealed container (*i.e.* allowing volatiles venting during the heating process); (iii) anaerobically burned in a sealed chamber. These are hereafter designated *A*, *An*_*unsealed*_ and *An*_*sealed*_, respectively.

### FTIR-ATR spectroscopy

FTIR-ATR data was recorded, for the powdered bone samples, in a Bruker Optics Vertex 70 FTIR spectrometer purged by CO_2_-free dry air and a Bruker Platinum ATR single reflection diamond accessory. A liquid nitrogen-cooled wide band mercury cadmium telluride (MCT) detector and a Ge on KBr substrate beamsplitter were used for the mid-IR interval (400–4000 cm^−1^). A room temperature deuterated L-alanine-doped triglycine sulfate (DLaTGS) detector with a polyethylene window and a Si beamsplitter were used for the far-IR range (50–600 cm^−1^).

128 scans were summed for each spectrum, at 2 cm^−1^ resolution, applying the 3-term Blackman–Harris apodization function, yielding a wavenumber accuracy above 1 cm^−1^. The Bruker OPUS—Spectroscopy Software (8.1 version)^42^ was used to correct the spectra regarding the wavelength dependence of the penetration depth of the electric field in ATR, for a mean refractive index of 1.25.

### Raman spectroscopy

Raman spectra were obtained for the powdered bone samples, in a WITec Raman microscope system alpha300R, coupled to an ultra-high throughput spectrometer 300 VIS grating (f/4 300 mm focal distance, 600 groves *per* millimetre blazed for 500 nm). The detection system was a 1650 × 200 pixels thermoelectrically cooled (− 55 °C at room temperature) charge-coupled device camera, front-illuminated with NIR/VIS antireflection coating, with a spectral resolution < 0.8 cm^−1^/pixel. The excitation radiation used was a 532 nm line of a frequency doubled Nd:YAG laser (*ca.* 10 mW at the sample position was applied). A 100 × objective (Zeiss Epiplan, NA 0.80, WD 1.3 mm) was used. 10 accumulations were collected *per* sample, with 30 s exposure time.

Autofluorescence is a property of bone tissue^43^ and fluorescent aromatic compounds may be formed during anaerobic burning^14,21^. This complicates Raman acquisition as fluorescence often masks the Raman signals, mainly for samples not subject to heat or burned at lower temperatures. This problem is particularly relevant for the 532 nm excitation wavelength^14^, used throughout this study. Thus, Raman data could only be obtained for the samples heated above 800 °C, both aerobically and anaerobically (sealed).

### INS spectroscopy

The INS measurements were carried out at the ISIS Pulsed Neutron and Muon Source of the STFC Rutherford Appleton Laboratory (United Kingdom), using the time-of-flight, high resolution broad range spectrometers MAPS^44,45^ and TOSCA^44,46,47^.

In MAPS, three incident energies were used (968, 2024 and 5240 cm^−1^) in order to accurately observe all the bands from hydroxyapatite, in both the low and high frequency ranges (the OH libration, its overtones and the OH stretching mode).

The samples (2 to 5 g) were wrapped in aluminium foil and fixed onto 4 × 4 cm thin walled aluminium cans. To reduce the impact of the Debye–Waller factor (the exponential term in Eq. (1)) on the observed spectral intensity, the samples were cooled to 5–10 K.

Data were recorded in the energy range 0 to 6000 cm^−1^ (MAPS) and 0 to 4000 cm^−1^ (TOSCA), and converted to the conventional scattering law, S(Q,ν) *vs* energy transfer (in cm^−1^) using the MANTID program (version 4.0.0)^48^. A spectrum was measured for an empty 4 × 4 cm thin walled aluminium can and subtracted from the data obtained for each bone sample.

## Results and discussion

Human femur (F) and humerus (H) were analysed upon aerobic and anaerobic burning. The samples were collected from the same skeleton, to avoid inter-skeleton variability^38^. The temperature range (400 to 1000 $^\circ$C) was chosen according to that reached in fire and explosion settings^49^, cremations^19^ and criminal burning of corpses^5^, allowing us to probe relevant heat-degradation events such as water and carbonate loss, destruction of the organic components, variations in crystal size and crystal rearrangements. No noteworthy variations were detected between the vibrational profiles of the two types of bones (femur and humerus), for each experimental condition under study.

Three different settings were applied for the burning experiments: (i) combustion, in the presence of oxygen (oxidising conditions) (*A*); (ii) reductive conditions (absence of oxygen), volatiles being continuously pumped out (*An*_*unsealed*_); (iii) oxygen-deprived environment (after vacuum pumping), in a sealed chamber (*An*_*sealed*_) not allowing the release of the volatiles formed during the burning process, which enabled a re-equilibrium to be attained. While in experiment (ii) the bone was powdered first and then burned, in (iii) the bone was burned as a whole fragment and powdered afterwards.

**Figure 2:**
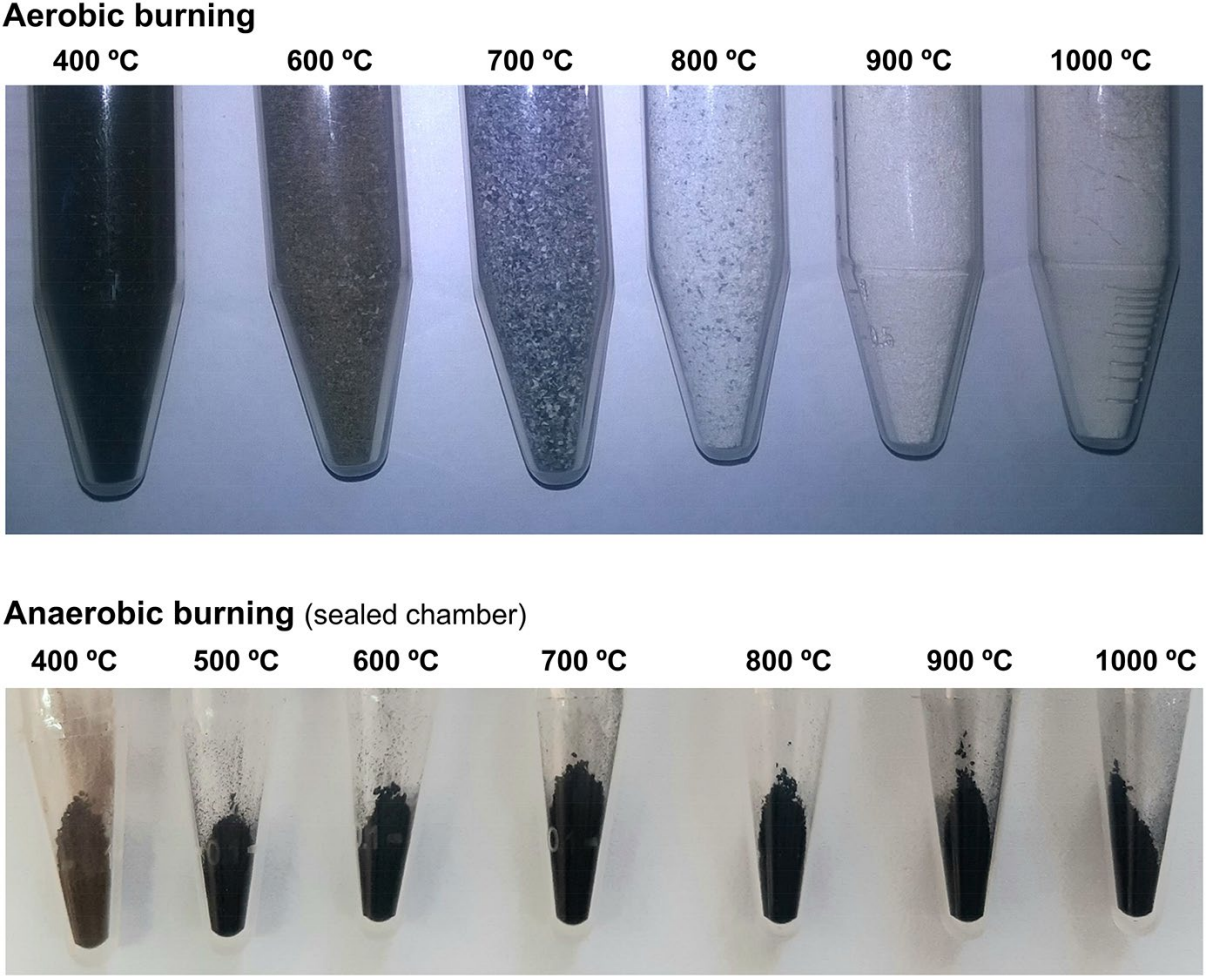
Human bone samples currently analysed, showing the colour changes according to the burning conditions—aerobic or anaerobic in a sealed chamber (An_sealed_).

Clear macroscopic differences were observed for the distinctly heated bones: the samples burned aerobically displayed a colour sequence with increasing temperatures from black (at 400 °C) to brown (at 500–600 °C) and white (> 800 °C, the typical colour for calcined bone); those heated anaerobically (either in sealed or unsealed chambers) were brown at the lowest temperature (400 °C) and consistently black above 450 °C (Fig. 2) (as previously verified by other authors^21,50^), due to the formation of amorphous inorganic carbon which is not produced in the presence of oxygen (when volatile CO_2_ is formed instead). These evident macroscopic differences already revealed a clearly distinct thermal alteration trajectory, as a function of temperature, for aerobically *versus* anaerobically burned bone.

### Heat-induced changes in bone´s inorganic matrix

**Figure 3:**
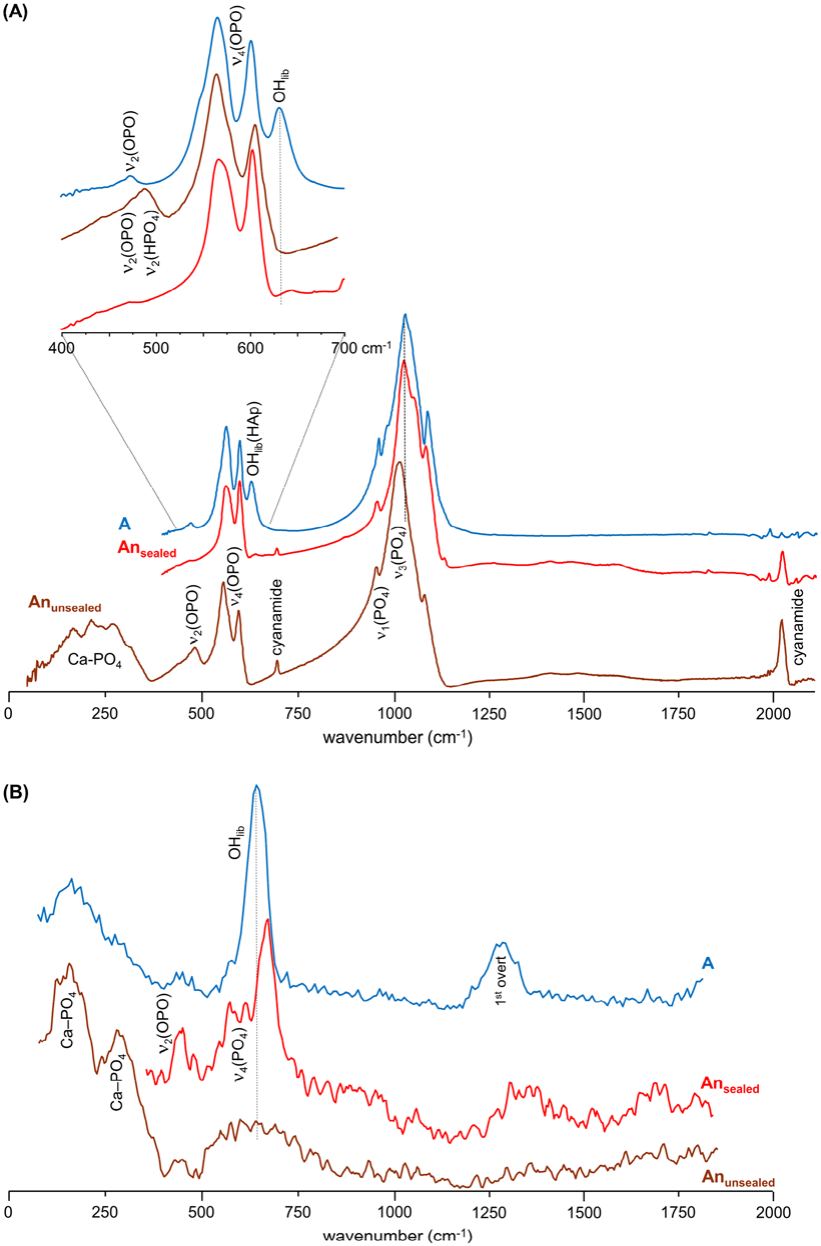
FTIR-ATR (**A**) and INS spectra (measured in MAPS, with 2024 cm^−1^ incident energy) (**B**) of human femur burned at 1000 °C under anaerobic/unsealed (An_unsealed_, brown), anaerobic/sealed (An_sealed_, red) and aerobic (A, blue) conditions.

**Figure 4:**
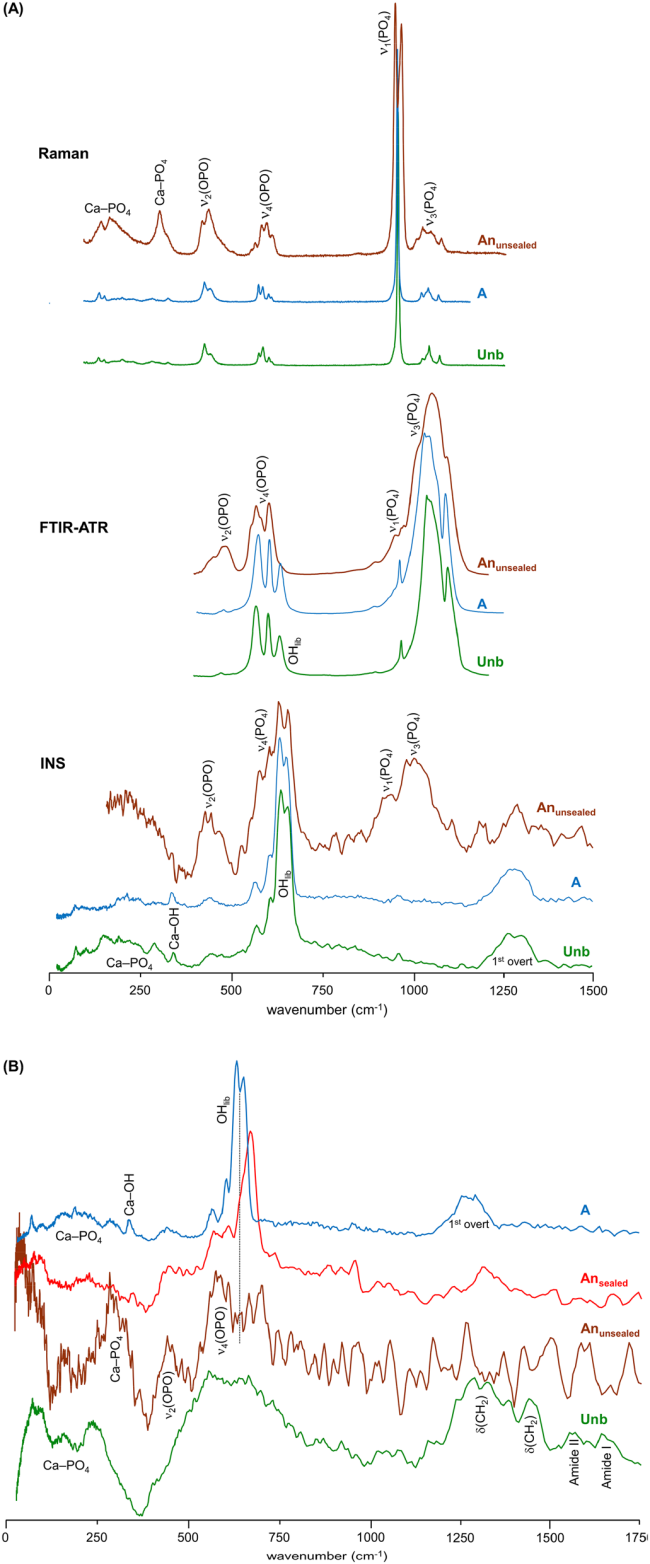
(**A**) INS (measured in TOSCA), FTIR-ATR and Raman spectra of reference calcium hydroxyapatite (SRM 2910b, HAp)—unburned (Unb, green), and burned at 1000 °C under aerobic (A, blue) and anaerobic/unsealed conditions (An_unsealed_, brown); (**B**) INS spectra (measured in TOSCA) for human femur, unburned (Unb, green) and burned at 1000 °C under anaerobic/unsealed (An_unsealed_, brown), anaerobic/sealed (An_sealed_, red) and aerobic (A, blue) conditions.

A significant chemical difference between the samples burned anaerobically in unsealed or in sealed containers was the formation of either graphitic carbon and Ca-phosphates (mainly β-tricalcium phosphate, Ca_3_(PO_4_)_2_^22,31^), or HAp at high temperatures, respectively. This was reflected in the corresponding infrared spectra (Fig. 3A) and even more clearly in the INS profiles (Figs. 3B, 4B and S1/Supplementary Information)—loss of hydroxyl groups was distinctly observed for the An_unsealed_ samples, while for the An_sealed_ bones the OH libration and stretching bands (at 660 and 3570 cm^−1^, respectively) were detected thanks to a re-equilibrium process that is only possible within a tightly closed environment (not allowing volatiles venting)—*i.e.* the hydroxyls may be incorporated back into the bone mineral yielding an inorganic framework very similar to HAp.

In turn, signals from the doubly degenerate v_2_(OPO) mode (with hydrogens close enough for it to be observed in INS) and HPO_4_^2-^ (at 451/483/578 cm^−1^), as well as from Ca-PO_4_ lattice vibrations (centered at 166 and 288 cm^−1^), were detected in the FTIR and INS spectra of An_unsealed_ samples at 1000 °C (Fig. 3A,B). Actually, the low wavenumber INS pattern currently obtained for An_unsealed_ femur and humerus at 1000 °C (Fig. 4B) was similar to the one assigned to brushite (CaHPO_4_·2H_2_O) by Taylor and co-workers^51^ (disregarding the bands from hydration water), who suggested that the HPO_4_^2−^ anion is in a brushite-like configuration in bone.

Hence, upon the loss of all the organic components of bone (lipids and collagen) through heating at high temperatures (900–1000 °C), the inorganic matrix was affected in different ways depending on the environmental conditions during the burning event: while OH librational and stretching modes from hydroxyapatite were observed above 600 °C for the samples burned aerobically or anaerobically under sealed conditions (blue shifted from 631 to 644 cm^−1^), they were found to disappear for the bones anaerobically heated in an unsealed chamber (with volatiles release).

The infrared and INS complementary vibrational profiles of reference HAp, at room temperature (unburned) and upon heating to 1000 °C in both aerobic and anaerobic unsealed conditions (Fig. 4A), evidenced a similar effect: the OH_lib_ infrared band virtually disappeared for the anaerobically burned HAp (with volatiles venting), while by INS (more sensitive to H-containing modes) it displayed a drastically reduced intensity—as reflected by the OH_lib_/ν_3_(PO_4_) and OH_lib_/ν_2_(OPO) ratios in the unburned *versus* An_unsealed_ samples. In turn, for HAp burned aerobically at the same temperature (1000 °C) the hydroxyl bands were clearly observed, yielding a profile very similar to that of unburnt hydroxyapatite. Moreover, the Raman profile of HAp anaerobically heated at 1000 °C showed a splitting of the ν_1_(PO_4_) signal which, coupled to the lower resolution observed for the other phosphate signals, is suggestive of the formation of more than one crystalline phase at this high temperature (hexagonal and monoclinic^51,52^). Additionally, an intensity variation was detected for the triply degenerate ν_4_(OPO) mode in the sample burned at 1000 °C in an aerobic setting as compared to the unburned bone (Fig. 4A), which is clearly indicative of a heat-induced crystallinity rearrangement.

The Raman spectra of both femur and humerus burned at 900 and 1000 °C corroborated the presence of hydroxyapatite in the An_sealed_ samples under these conditions—clearly displaying the characteristic signals from phosphate, at 426, 590, 960 and 1030 cm^−1^ (ν_2_(OPO), ν_4_(OPO), ν_1_(PO_4_) and ν_3_(PO_4_), respectively) (Fig. S2/Supplementary Information).

### Heat-induced changes in bone´s organic constituents

**Figure 5:**
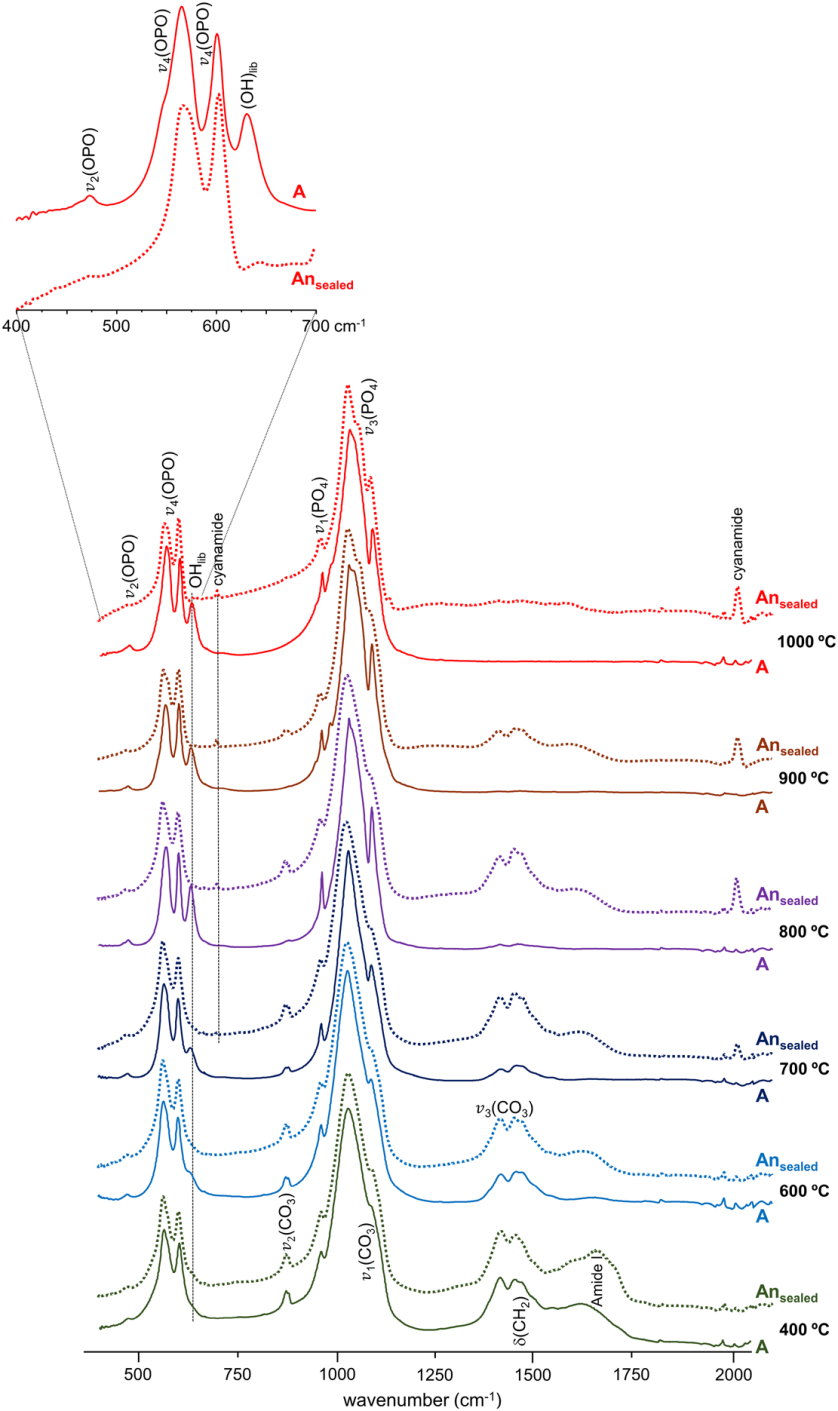
FTIR-ATR spectra (400–2000 cm^−1^) of human humerus burned at different temperatures (400 to 1000 ° °C) either under aerobic (**A**) or anaerobic/sealed (An_sealed_) conditions. The insert shows a magnification of the phosphate and OH librational bands from hydroxyapatite.

For the burning process in the absence of oxygen (in sealed containers) the loss of bone´s organic constituents appeared to be slowed down as compared to aerobic burning, reflecting a somewhat retarded heat effect on these components—lipids and proteins (mainly collagen type I). Actually, the infrared CH_2_ deformation modes from the lipids and peptide carbon chains (1400–1450 cm^−1^) and the characteristic protein signal at 1650 cm^−1^ (ν(C=O), Amide I) were found to disappear at *ca.* 600 °C in aerobic conditions (due to combustion of these organic constituents), but were still observed at temperatures as high as 800 °C in anaerobic (sealed) settings (Fig. 5). Also, the typical ν_2_(CO_3_)_A_ and ν_3_(CO_3_) carbonate features around 870 and 1420 cm^−1^ (the latter contributing to the broad signal detected in the 1400–1500 cm^−1^ interval) were detected even at 900 °C, disappearing completely only at 1000 °C. Since no venting of the volatiles was possible in the closed containers where burning took place, these observations are probably due to a re-equilibrium of the CO_2_ released upon heating that led to a reintroduction of carbonates into the bone´s framework.

**Figure 6:**
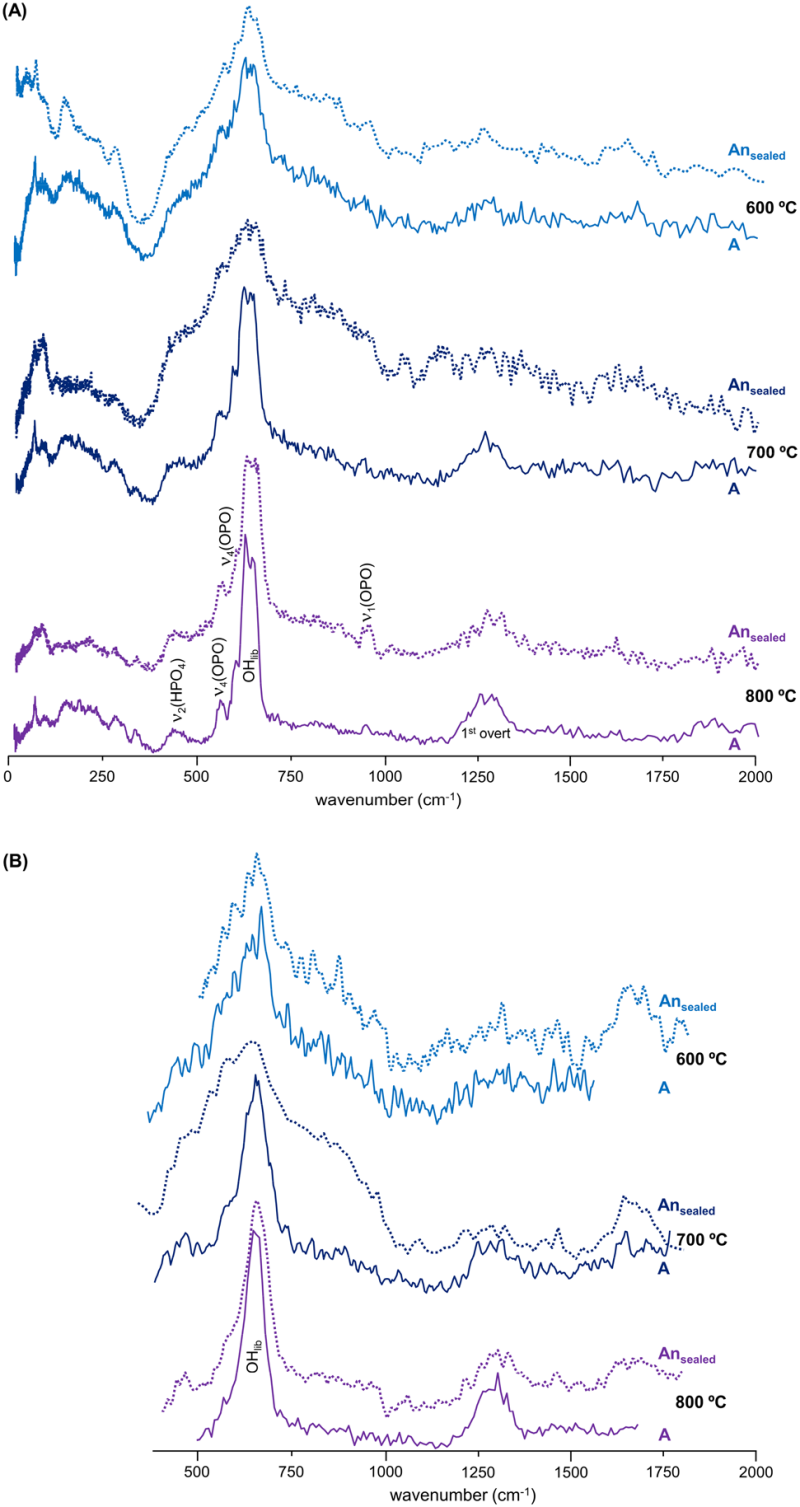
INS spectra of human femur burned at different temperatures (600 to 800 °C) either under aerobic (**A**) or anaerobic/sealed (An_sealed_) conditions. (**A**) Data measured in TOSCA; (**B**) Data measured in MAPS (with 2024 cm^−1^ incident energy).

Figures 6 and S3 (Supplementary Information) comprise the INS profiles of human femur burned aerobically and anaerobically (in sealed chambers)—measured in TOSCA and in MAPS, thus allowing all bands within the 0–2000 cm^−1^ spectral window to be detected with high sensitivity. The samples burned anaerobically (An_sealed_) yielded a less defined profile as compared to those heated aerobically, probably because for the latter the volatiles formed upon heating were continuously vented during the process as opposed to the burning experiments inside an airtight container. Also, while for the aerobically burned samples the main spectral differences were detected between 600 and 700 °C (as previously reported for other types of bones^13^), for those heated in the absence of oxygen in a sealed environment the major variations occurred at higher temperatures (from 700 to 800 °C), in accordance with the delayed heat effect on bone´s organic components under reductive conditions (mostly revealed by FTIR). As temperature increased, the disorganisation of the bone structure triggered by loss of the organic constituents (mainly collagen), reflected in a marked broadening of the INS bands, appeared to be followed by an organisation of the inorganic matrix, thus justifying the narrow and well defined signals observed at 800 °C (and above).

### Formation of cyanamide and portlandite

Another striking difference between the bones burned under aerobic or anaerobic conditions was the formation of cyanamide (NCNH_2_) for the latter, both in sealed and unsealed environments, at temperatures above 700 °C (probably at the surface of the bone samples). This was revealed by two characteristic features at 700 and 2009 cm^−1^, ascribed to NCN deformation and stretching modes, respectively (Figs. 5 and 3A). Although cyanamide may be due to soil contamination (since calcium cyanamide is a common agricultural fertilizer), its incorporation into the bone matrix was also previously proposed to occur during heating processes under reducing conditions, associated with an incomplete oxidation of organic matter^21,27,50^—cyanamide substituting for hydroxyl groups within the apatite framework (instead of type A carbonates), one cyanamide replacing *ca.* 2.3 OH´s^27^. Therefore, the presence of cyanamide bands in the infrared spectrum, concomitant with the absence of hydroxyapatite´s OH signals, can be used to identify burning processes in oxygen-deprived environments, both in forensic and archaeologic settings.

Furthermore, a peak assigned to the OH stretching mode of portlandite (the naturally occurring form of calcium hydroxide, Ca(OH)_2_)^53,54^ was clearly visible at *ca.* 3640 cm^−1^ for the bones burned anaerobically in unsealed conditions (Fig. S1/Supplementary Information). This signal, previously observed by other authors in bones burned at high temperatures^39,54^, was presently not detected for the aerobically heated samples nor for those anaerobically burned in sealed containers. It is therefore suggested that portlandite´s ocurrence in these samples may be due to the decomposition of hydroxyapatite at very high temperatures, in anaerobic environments allowing volatiles venting. This is corroborated by the variations observed in the low frequency region of the corresponding INS profiles (measured in TOSCA with very high sensitivity), in accordance with the rearrangements within bone´s inorganic framework particularly regarding the Ca–PO_4_ and Ca–OH lattice modes (Fig. 4 (B)).

### Quantitative effect of the burning process on bone´s vibrational profile

**Figure 7:**
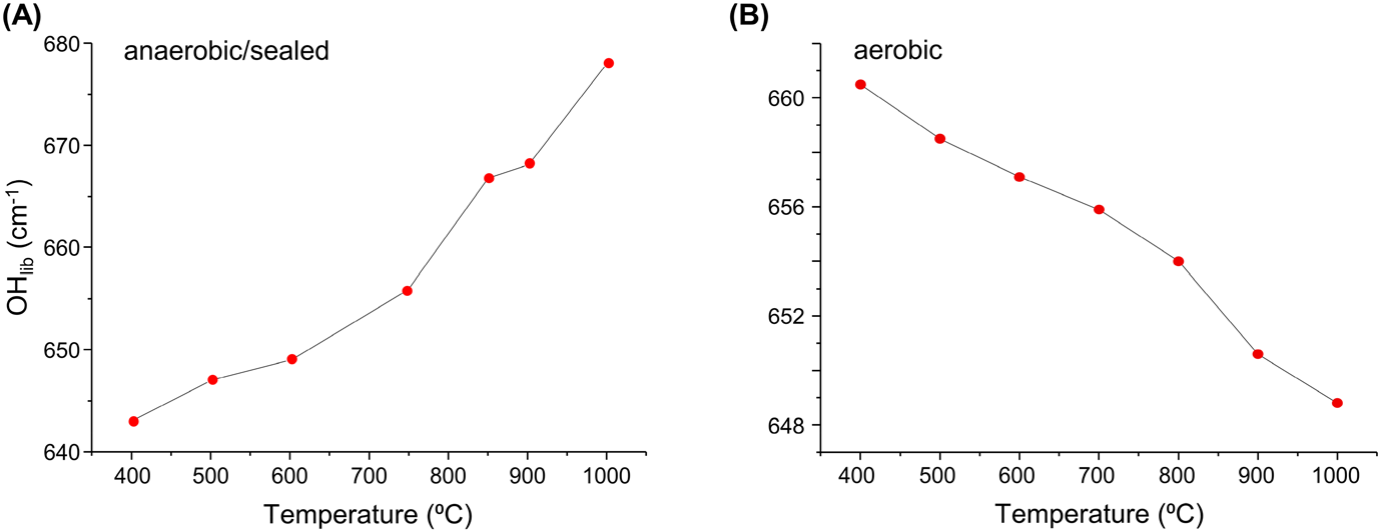
Temperature dependence of the INS hydroxyapatite´s hydroxyl libration for human femur: (**A**) burned under anaerobic/sealed conditions (data measured in MAPS, with 2024 cm^−1^ incident energy); (**B**) burned under aerobic conditions (data measured in TOSCA).

The temperature dependence of HAp´s hydroxyl libration wavenumber measured for the An_sealed_ samples evidenced a blue shift with increasing temperature – Δ  = 35 cm^−1^, from 643 cm^−1^ at 400 °C to 678 cm^−1^ at 1000 °C. This behaviour is opposed to that previously identified by the authors for similar samples burned aerobically^13^, which displayed a less marked and opposite shift (red shift of *ca.* 12 cm^−1^) of this librational mode upon heating (Figs. 7 and S3/Supplementary Information).

Furthermore, while for the aerobically burned samples there is probably a resonance between the HAp´s libration and the ν_4_(OPO) mode, under anaerobic conditions a different polymorphic structure is suggested to be formed, leading to a blue shift of HAp_lib_ and the disappearance of the resonance effect thus justifiying the very low intensity of the corresponding band. The formation of distinct bioapatite polymorphs for bones heated anaerobically at very high temperatures is in agreement with the splitting observed for the corresponding ν_1_(PO_4_) signal (previously discussed).

Table 1 comprises the main variations detected by vibrational spectroscopy (Raman, FTIR and INS) for burned human bones burned under different settings—aerobic, anaerobic/unsealed and anaerobic/sealed conditions.

**Table 1:** Main changes detected by vibrational spectroscopy (FTIR-ATR, Raman and INS) in samples from human bones burned under different conditions—aerobic, anaerobic/sealed chamber and anaerobic/unsealed chamber.

Vibrational band	Wavenumber (cm^−1^)	Detected by	Burning conditions		
			Aerobic	Anaerobic	
				Unsealed (1000 °C)	Sealed
τ(CH_3_)_protein_	250	INS	Not detected	Not detected	Up to 400 °C
(OH)_HAp-lib_	630–680	FTIR, INS	> 600 °C	Not detected	> 400 °C
δ(CNC)_cyanamide_	700	FTIR	Not detected	Detected	> 800 °C
ν_2_(CO_3_)_A_	870	FTIR	Up to 700 °C	Not detected	Up to 900 °C
ν_3_(CO_3_)	*ca.* 1420	FTIR	Up to 700 °C	Not detected	Up to 900 °C
δ(CH_2_)_lipids_	*ca.* 1450	FTIR, INS	Up to 700 °C	Not detected	Up to 900 °C
δ(C = O)_protein_	1650	FTIR, INS	Up to 600 °C	Not detected	Up to 900 °C
ν(CN)_cyanamide_	2009	FTIR	Not detected	Detected	> 700 °C
ν(OH)_HAp_	*ca.* 3580	FTIR, Raman, INS	Detected	Not detected	Detected
ν(OH)_portlandite_	3640	FTIR	Not detected	Detected	Not detected

## Conclusions

Complementary vibrational spectroscopic data was measured for human bones burned under different conditions regarding oxygen availability, for a wide temperature range (up to 1000 °C): combustion (in the presence of oxygen), yielding hydroxyapatite at the highest temperatures; absence of oxygen, for two distinct underlying processes—producing either graphitic carbon and Ca-phosphates (with volatiles venting) or HAp (in a sealed container). This delivered accurate information regarding the heat-induced variations on the bone´s organic and inorganic matrices, as a function of the environmental conditions, by assessing changes within the samples subject to increasing temperatures, while water, organic components (lipids and proteins) and carbonates were gradually driven out—thus characterising bone´s crystalline framework at well-defined temperatures.

Overall, a burning process at high temperatures under anaerobic conditions leads to a decrease of the hydroxyl amount within the bone matrix (also observed for standard hydroxyapatite), which is much more pronounced when the heating occurs in an unsealed chamber—allowing evacuation of the volatile products formed during the process and thus hindering re-equilibrium of the hydroxyl groups into the bone´s inorganic framework (with the reappearance of the characteristic OH libration and stretching modes). The absence of HAp´s typical OH vibrational bands (mainly the libration), coupled to the presence of cyanamide and portlandite, were found to be good indicators of bone burning under anaerobic conditions in an unsealed environment. These are thus reliable spectroscopic biomarkers prone to be used in real scenarios for the identification of burning conditions of human skeletal remains from both forensic and archaeological settings. Furthermore, the disappearance of the hydroxyl signals from bone´s inorganic framework may also be ascribed to fossilisation processes occurring under reducing conditions, in accordance with a previous study by the authors on bone fossil samples for which no OH bands were observed (despite their high crystallinity)^33^.

The results presently gathered complement previous data obtained by the team on human bones burned under aerobic conditions, through FTIR, Raman and INS. This combined approach provided an improved understanding of the chemical and microcrystallinity variations undergone by bone when subject to heating, either under oxidising or reducing conditions. This is an innovative way of tackling structural and chemical transformations in bone, which is expected to have a high impact in forensic, bioanthropological and archaeological sciences. Concerning forensic human skeletal remains subject to intense heating (rendering DNA analysis virtually impossible), there is a wide range of possible scenarios—from house fires, vehicle accidents or bomb attacks, to homicides where the victim´s body is cremated in order to conceal evidences. Regarding archaeological settings, the present study is expected to contribute to a full characterisation of ancient burned bones, particularly regarding the identification of the specific heating conditions (*e.g.* oxygen availability) to which they were subject.

## Supplementary Information

Supplementary Figures.

## Data Availability

The data that support the findings of this study are available from the corresponding author upon reasonable request.
