# Supplementary material for: Profiling of human burned bones: oxidising versus reducing conditions
Source: Sci Rep. 2021 Jan 14;11:1361. doi: 10.1038/s41598-020-80462-3 (PMC7809265; doi:10.1038/s41598-020-80462-3)
Supplement: Supplementary file 1 — Supplementary Figures. [file 41598_2020_80462_MOESM1_ESM.pdf]

Supplementary Information

Profiling of Human Burned Bones: Oxidising *versus* Reducing Conditions

M.P.M. Marques[1,2], D. Gonçalves[3,4,5], A.P. Mamede[1], T. Coutinho[2], E. Cunha[2,3], W. Kockelmann[6], S.F. Parker[6] & L.A.E. Batista de Carvalho[1*]

[1]University of Coimbra, “Molecular Physical Chemistry” R&D Unit, Department of Chemistry, 3004-535 Coimbra, Portugal
[2]University of Coimbra, Department of Life Sciences, 3000-456 Coimbra, Portugal
[3]University of Coimbra, Laboratory of Forensic Anthropology, Centre for Functional Ecology, 3000-456 Coimbra, Portugal
[4]University of Coimbra, Research Centre for Anthropology and Health (CIAS), 3000-456 Coimbra, Portugal
[5]Archaeosciences Laboratory, Directorate General Cultural Heritage (LARC/CIBIO/InBIO), 1349-021 Lisbon, Portugal
[6]ISIS Facility, STFC Rutherford Appleton Laboratory, Chilton, Didcot, OX11 0QX, United Kingdom

Table of Contents

Figure S1 – Raman, FTIR-ATR and INS (measured in MAPS with 5240 cm^{-1} incident energy) spectra of human femur burned at 1000 ºC under aerobic (A, blue), anaerobic/unsealed ($An_{unsealed}$, brown) or anaerobic/sealed conditions (An_{sealed}, red). The INS spectrum of unburn reference calcium hydroxyapatite (SRM 2910b, HAp) is shown for comparison.

Figure S2 – Raman spectra of human femur and humerus burned at 1000 ºC under anaerobic/sealed conditions (An_{sealed}), and for reference calcium hydroxyapatite (SRM 2910b, HAp) at room temperature.

Figure S3 – INS spectra of human femur – unburned, and burned at different temperatures (400 to 1000 ºC) under anaerobic/sealed (An_{sealed}) conditions. (A) Data measured in TOSCA; (B) – Data measured in MAPS (with 2024 cm^{-1} incident energy).

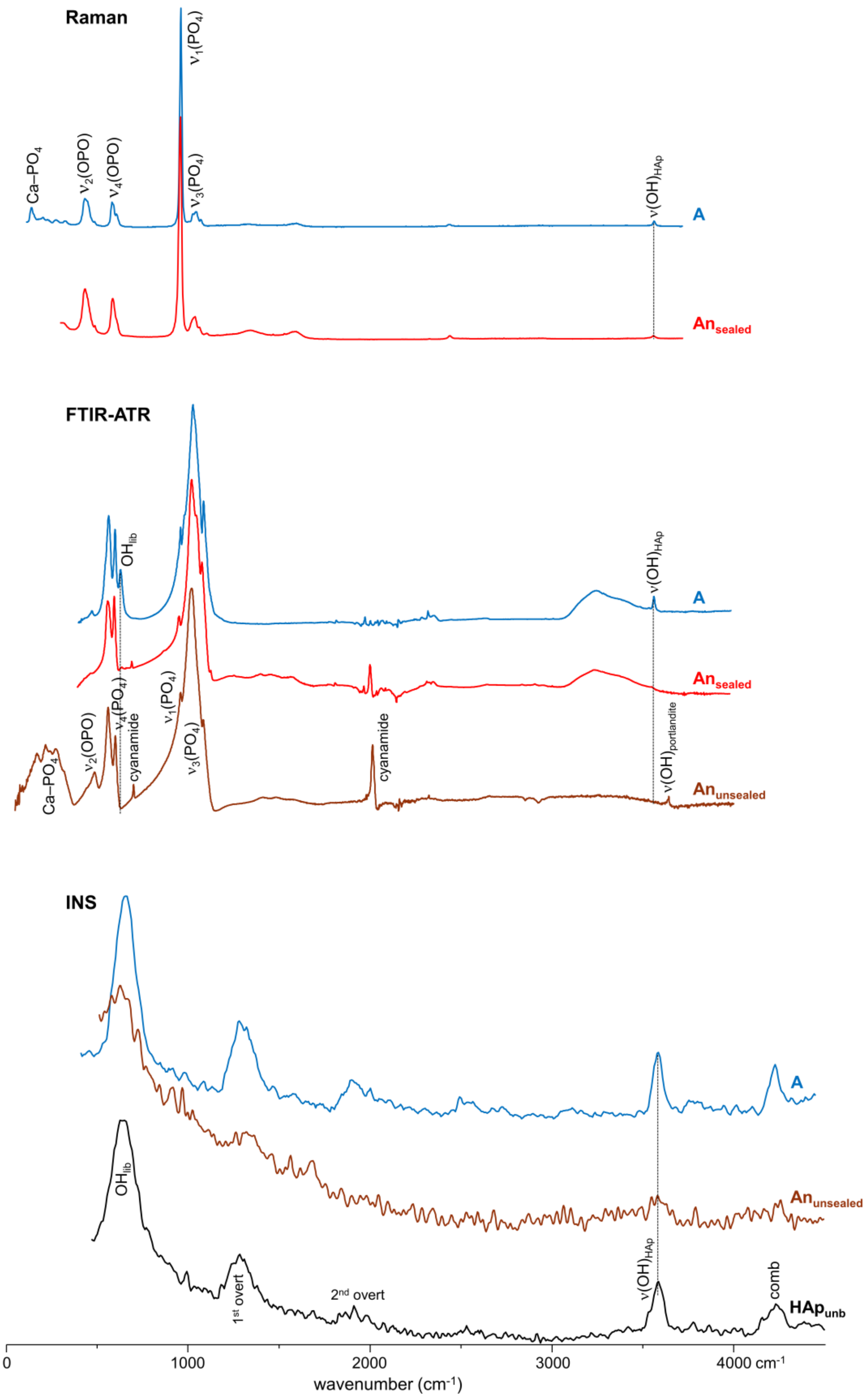

Figure S1 – Raman, FTIR-ATR and INS (measured in MAPS with 5240 cm^{-1} incident energy) spectra of human femur burned at 1000 ºC under aerobic (A, blue), anaerobic/unsealed ($An_{unsealed}$, brown) or anaerobic/sealed (An_{sealed}, red) conditions. The INS spectrum of unburn reference calcium hydroxyapatite (SRM 2910b, HAp) is shown for comparison.

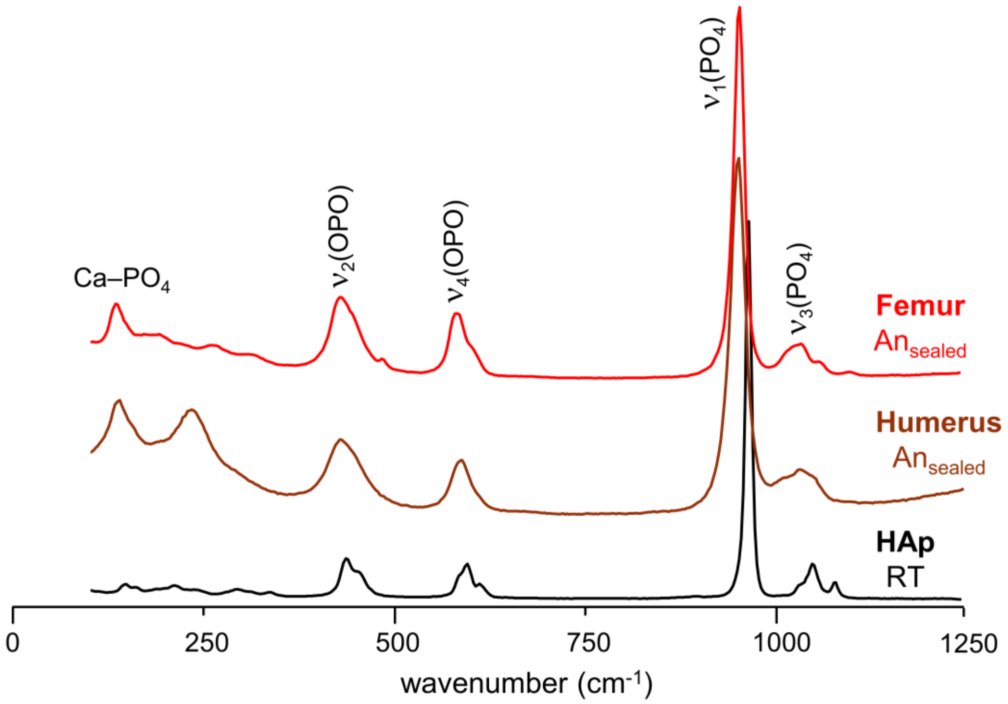

Figure S2 – Raman spectra of human femur and humerus burned at 1000 ºC under anaerobic/sealed conditions (An_{sealed}), and for reference calcium hydroxyapatite (SRM 2910b, HAp) at room temperature.

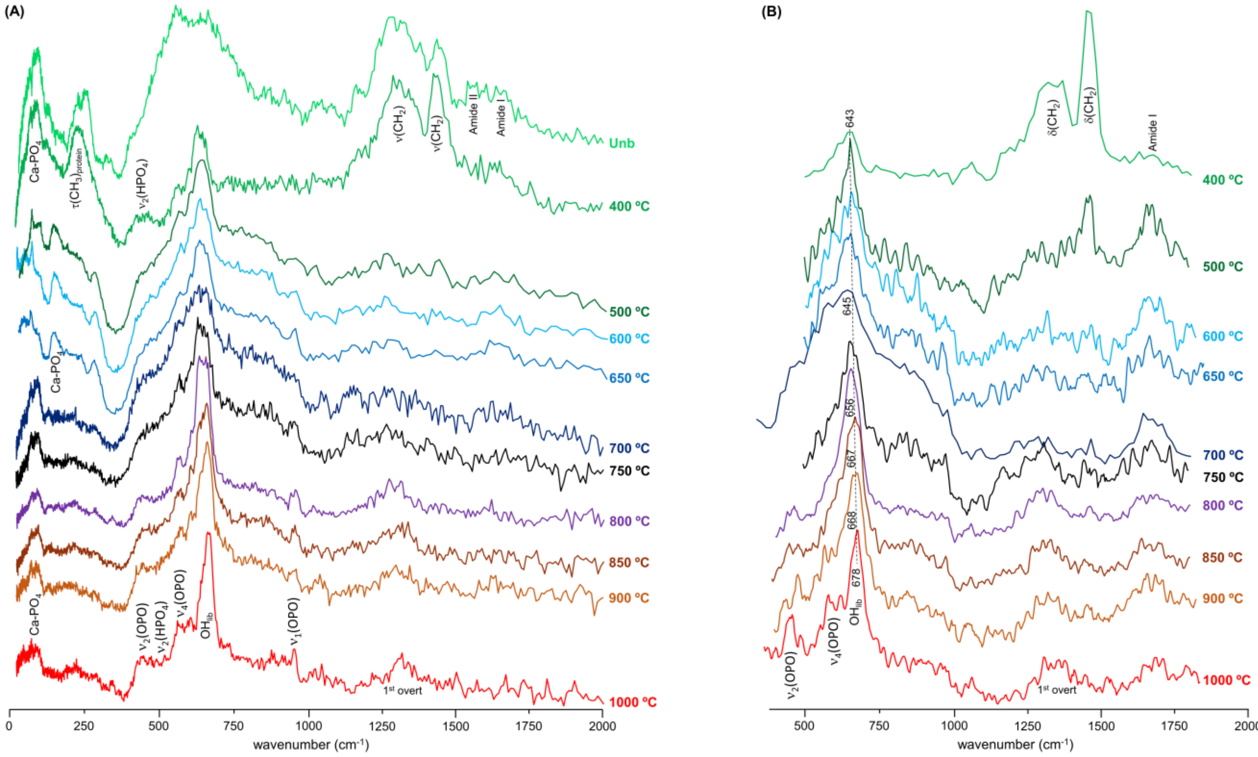

Figure S3 – INS spectra of human femur – unburned, and burned at different temperatures (400 to 1000 ºC) under anaerobic/sealed (An_{sealed}) conditions. (A) Data measured in TOSCA; (B) – Data measured in MAPS (with 2024 cm^{-1} incident energy).
